# CYP46A1-mediated cholesterol turnover induces sex-specific changes in cognition and counteracts memory loss in ovariectomized mice

**DOI:** 10.1126/sciadv.adj1354

**Published:** 2024-01-24

**Authors:** María Latorre-Leal, Patricia Rodriguez-Rodriguez, Luca Franchini, Orestis Nikolidakis, Makrina Daniilidou, Ljerka Delac, Mukesh K. Varshney, Luis E. Arroyo-García, Francesca Eroli, Bengt Winblad, Kaj Blennow, Henrik Zetterberg, Miia Kivipelto, Manuela Pacciarini, Yuqin Wang, William J. Griffiths, Ingemar Björkhem, Anna Matton, Ivan Nalvarte, Paula Merino-Serrais, Angel Cedazo-Minguez, Silvia Maioli

**Affiliations:** ^1^ Department of Neurobiology Care Sciences and Society, Division of Neurogeriatrics, Center for Alzheimer Research, Karolinska Institutet, Stockholm, Sweden.; ^2^ Department of Neurobiology Care Sciences and Society, Division of Clinical Geriatrics, Center for Alzheimer Research, Karolinska Institutet, Stockholm, Sweden.; ^3^ Department of Biosciences and Nutrition, Karolinska Institutet, Huddinge, Sweden.; ^4^ Institute of Neuroscience and Physiology, University of Gothenburg, Mölndal, Sweden.; ^5^ Clinical Neurochemistry Laboratory, Sahlgrenska University Hospital, Mölndal, Sweden.; ^6^ Institut du Cerveau et de la Moelle épinière (ICM), Pitié-Salpêtrière Hospital, Sorbonne Université, Paris, France.; ^7^ University of Science and Technology of China, Hefei, Anhui, P.R. China.; ^8^ Theme Aging, Karolinska University Hospital, Stockholm, Sweden.; ^9^ Swansea University Medical School, SA2 8PP Swansea, UK.; ^10^ Department of Laboratory Medicine, Karolinska Institutet, Huddinge, Sweden.; ^11^ Departamento de Neurobiología Funcional y de Sistemas, Instituto Cajal, CSIC, Madrid, Spain.; ^12^ Laboratorio Cajal de Circuitos Corticales, Centro de Tecnología Biomédica, UPM, Madrid, Spain.

## Abstract

The brain-specific enzyme CYP46A1 controls cholesterol turnover by converting cholesterol into 24*S*-hydroxycholesterol (24OH). Dysregulation of brain cholesterol turnover and reduced *CYP46A1* levels are observed in Alzheimer’s disease (AD). In this study, we report that *CYP46A1* overexpression in aged female mice leads to enhanced estrogen signaling in the hippocampus and improved cognitive functions. In contrast, age-matched *CYP46A1* overexpressing males show anxiety-like behavior, worsened memory, and elevated levels of 5α-dihydrotestosterone in the hippocampus. We report that, in neurons, 24OH contributes to these divergent effects by activating sex hormone signaling, including estrogen receptors. *CYP46A1* overexpression in female mice protects from memory impairments induced by ovariectomy while having no effects in gonadectomized males. Last, we measured cerebrospinal fluid levels of 24OH in a clinical cohort of patients with AD and found that 24OH negatively correlates with neurodegeneration markers only in women. We suggest that CYP46A1 activation is a valuable pharmacological target for enhancing estrogen signaling in women at risk of developing neurodegenerative diseases.

## Teaser

*CYP46A1* overexpression is neuroprotective in female mice during chronological and endocrine aging.

## INTRODUCTION

The neuron-enriched enzyme cholesterol 24-hydroxylase (CYP46A1) promotes cholesterol excretion from the brain by converting cholesterol into its oxidized metabolite, 24*S*-hydroxycholesterol (24OH) (*1*). A constant cholesterol flux into 24OH is essential for brain function, including learning and memory (*2*). *Cyp46a1* knockout mice display memory deficits (*3*), while overexpression of human *CYP46A1* in older females results in increased levels of synaptic proteins and better performance in spatial memory tasks (*4*). On the other hand, some evidence reported that up-regulation of *CYP46A1* may negatively affect aging processes, where stress conditions and 24OH levels above physiological concentrations could lead to CYP46A1 hyperactivity and cholesterol loss (*5*–*7*).

Brain cholesterol metabolism is altered already at the early stages of Alzheimer’s disease (AD) and the levels of the oxysterol 24OH are modified in both plasma and cerebrospinal fluid (CSF) from patients with AD (*8*–*11*). Recent findings showed a reduction of *CYP46A1* and 24OH levels in AD brains (*12*, *13*), and increasing evidence suggests that *CYP46A1* activation may prevent pathological processes occurring in AD (*14*, *15*). Injections of adeno-associated virus encoding *CYP46A1* in the hippocampus of AD mouse models reduced amyloid β (Aβ) burden and restored spatial memory performance (*16*, *17*), while *Cyp46a1* inhibition led to opposite effects (*18*). These studies were performed only in female mice. Pharmacological activation of CYP46A1 with efavirenz in male and female AD mouse models ameliorated spatial memory (*19*) and familial human AD induced pluripotent stem cell (iPSC)–derived neurons treated with efavirenz showed decreased Aβ secretion and Tau phosphorylation (*20*). In addition to AD, *CYP46A1* activation revealed neuroprotective effects in Huntington’s disease (*20*) and Parkinson’s disease (*21*).

Besides the role of CYP46A1 as a regulator of neuronal cholesterol homeostasis, its metabolic product 24OH is a signaling molecule that affects brain metabolism and synaptic function by binding to liver X receptors (LXRs), retinoic acid–related orphan receptor (RORα and RORγ) (*22*), and *N*-methyl-d-aspartate receptor (NMDAR) (*23*, *24*). LXR activation promotes the synthesis of neuroactive steroids in the brain (*25*), including estrogen and testosterone. These sex hormones exert a range of neurotrophic effects on the brain by modulating pathways essential for neuronal plasticity, neurodevelopment, and behavior (*26*–*29*).

Particularly in women, early menopause, whether spontaneous or caused by ovarian removal, chemotherapy, or aromatase inhibitor treatment, is a risk factor for cognitive decline and higher levels of AD neuropathology (*30*–*34*). This suggests that sex hormones may have a different impact on AD pathophysiology and progression in men and women. It is known that more women suffer from AD (*35*) and present a faster disease progression than men (*36*). Moreover, women carrying *APOE4* run a fourfold higher risk of AD than male carriers (*37*). It is, therefore, possible that maintenance of cholesterol homeostasis is especially relevant for females; however, the mechanisms underlying sex differences in AD pathogenesis are still largely unexplored (*38*).

Given the relevance of CYP46A1 and the possible interplay between brain cholesterol metabolism and sex hormones in AD, we aimed to investigate the sex-specific effects of *CYP46A1* overexpression and 24OH in cognitive functions during chronological and endocrine aging in vivo and in vitro. Last, we performed sex-stratified analyses to test the association between CSF 24OH levels and markers of neurodegeneration in a memory clinic cohort of patients with AD, mild cognitive impairment (MCI), and subjective cognitive impairment (SCI).

## RESULTS

### *CYP46A1* overexpression improves memory performance only in aged female mice

**Fig. 1.**
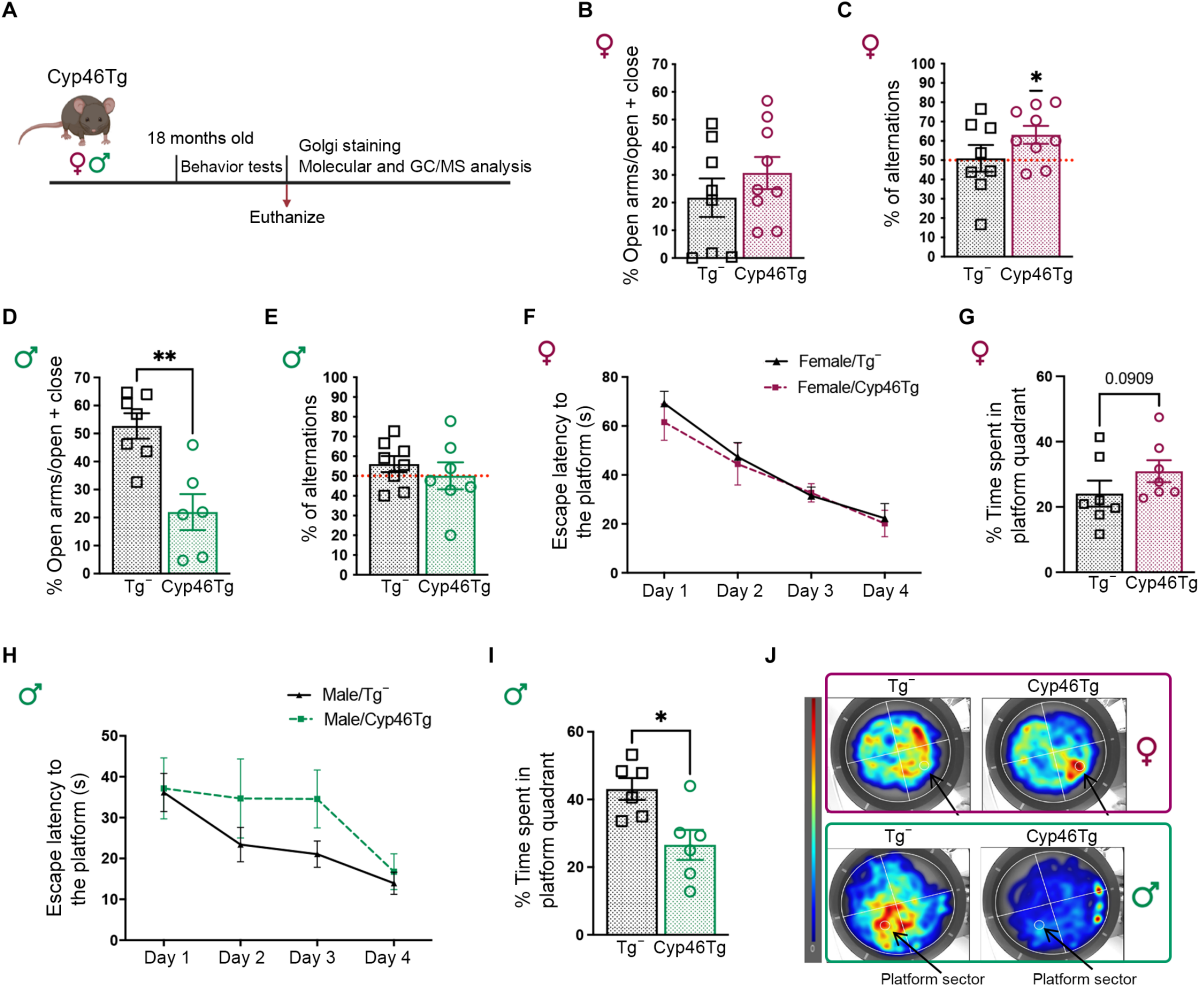
Behavioral tests in aged Cyp46Tg mice. (**A**) Experimental design: Aging female and male Cyp46Tg mice were assessed by elevated plus maze (EPM), Y-maze, and Morris water maze (MWM) tests. After the behavioral tests, the brains were collected for further molecular analysis. The experimental design was created with Biorender.com. (**B**) Percentage of time spent in the open arms during the EPM test in Cyp46Tg and Tg^−^ females. (**C**) Percentage of spontaneous alternations during the Y-maze test in female Cyp46Tg and Tg^−^ mice (one-sample *t* test, *P* = 0.022). (**D**) EPM test represented as the percentage of time spent in open arms in Cyp46Tg and Tg^−^ males (unpaired *t* test, *P* = 0.0047). (**E**) Percentage of spontaneous alternations in Y-maze test in male Cyp46Tg and Tg^−^ mice. (**F**) Escape latency over 4 days acquisition phase in the MWM test in Cyp46Tg and Tg^−^ females [repeated-measures analysis of variance (ANOVA), the effect of days *P* = 0.0001]. Adapted from data previously shown in (*4*). (**G**) Time spent in the platform quadrant during the probe test in Cyp46Tg and Tg^−^ females (unpaired *t* test, *P* = 0.09). Adapted from data previously shown in (*4*). (**H**) Escape latency over the 4-day acquisition phase in the MWM test in Cyp46Tg and Tg^−^ males (repeated-measures ANOVA, the effect of days *P* = 0.0094). (**I**) Time spent in the platform quadrant during the probe test (unpaired *t* test, *P* = 0.0130). (**J**) Representative heatmaps of the MWM probe test in female and male Cyp46Tg and Tg^−^ mice. Red zones display the areas mostly explored by the mice. *N* = 6 to 10 mice per group per sex. Data are represented as means ± SEM. *P < 0.01 and **P < 0.001.

We performed a battery of behavioral tests to evaluate anxiety-like behavior and hippocampal-dependent learning and memory in 18-month-old *CYP46A1* overexpressing male and female mice (Cyp46Tg) and their age-matched wild-type littermates (Tg^−^) (Fig. 1A). During the elevated plus maze (EPM) test, Cyp46Tg females did not show differences in the time spent in open arms compared to the control group (Fig. 1B). When females were assessed for spatial working memory in the Y-maze, only Cyp46Tg mice alternated at higher levels than the chance level of 50% (Fig. 1C). Differently from females, Cyp46Tg males spent a shorter time in the open arms of the EPM compared to the Tg^−^ (Fig. 1D), while Y-maze testing did not show significant differences between male groups (Fig.1E). Spatial learning and memory were tested in the Morris water maze (MWM) test (Fig. 1, F to J). During the acquisition, both female and male groups learned to locate the hidden platform over the days (Fig. 1, F and H). During the probe test, the transgenic females occupied the platform sector substantially longer than their controls (Fig. 1, G and J), as previously shown in our study (*4*), while Cyp46Tg males spent significantly shorter time than Tg^−^ mice in the quadrant where the platform was located (Fig. 1, I and J). When younger, 9-month-old mice were tested for the EPM and the MWM, we did not find differences between groups and sexes (fig. S1, A to F).

### *CYP46A1* overexpression has positive effects on dendritic spine morphology in female mice but not in males

**Fig. 2.**
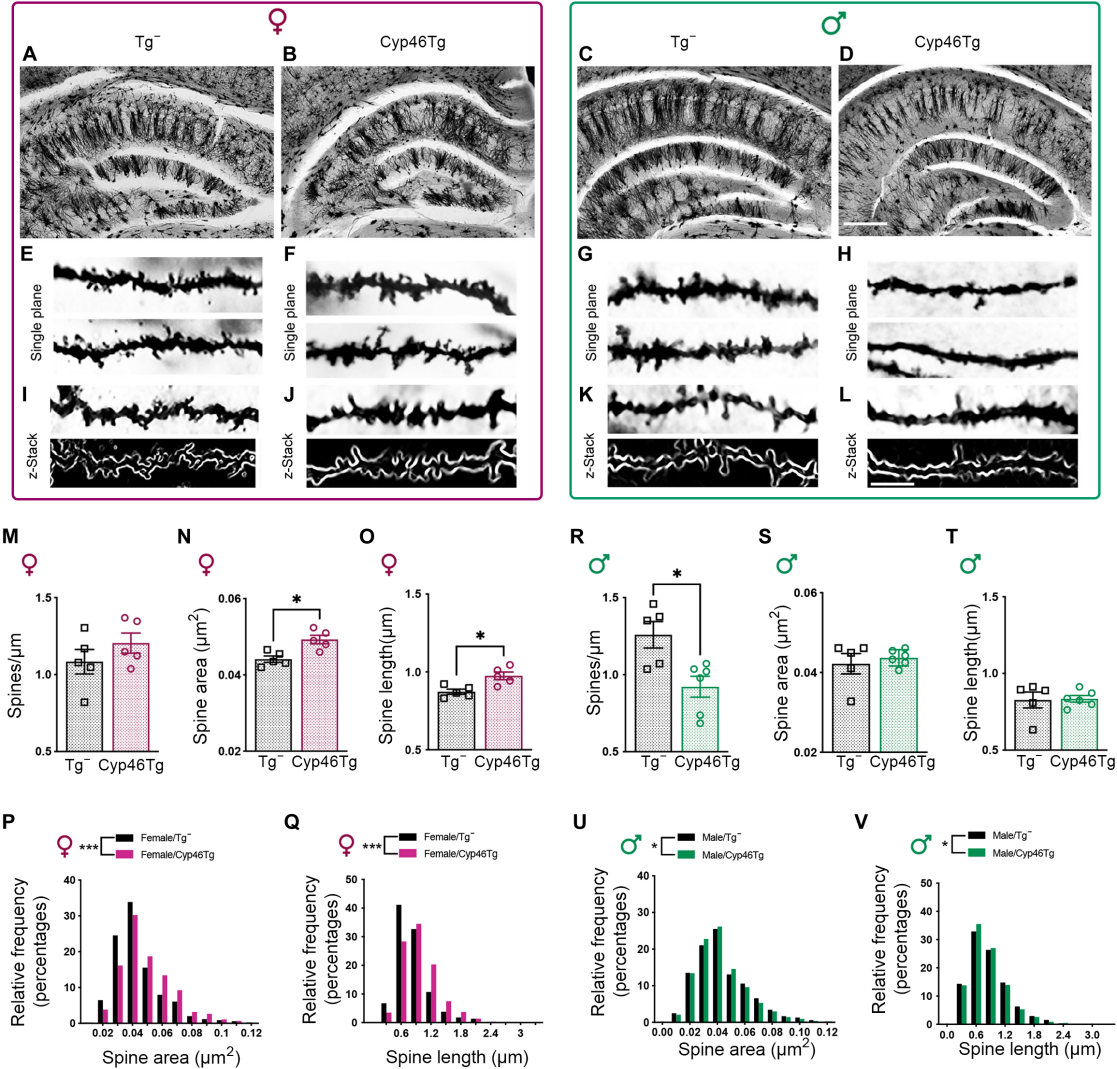
Golgi staining in the hippocampus of Cyp46Tg aged mice. (**A** to **L**) Representative Golgi staining images from stratum radiatum from the CA1 region of 18-month-old female (left) and male (right) Cyp46Tg mice and their corresponding age-matched Tg^−^ controls. Scale bars, 500 μm [(A) to (D)] and 5 μm [(E) to (L)]. (**M**) Analysis of dendritic spine density measured as spines per micrometer in female Cyp46Tg compared to Tg^−^ mice. (**N**) Spine area in square micrometer in Cyp46Tg and Tg^−^ females (unpaired *t* test, *P* = 0.0416). (**O**) Spine length in female Cyp46Tg and Tg^−^ mice (unpaired *t* test, *P* = 0.0384). (**P**) Frequency distribution histogram of spine area in female mice (two-sample Kolmogorov-Smirnov test, *P* < 0.0001). (**Q**) Frequency distribution histogram of spine length of female Cyp46Tg and Tg^−^ mice (two-sample Kolmogorov-Smirnov test *P* < 0.001). (**R**) Dendritic spine density in male Cyp46Tg and Tg^−^ mice (unpaired *t* test, *P* = 0.0122). (**S**) Spine area in male Cyp46Tg compared to male Tg^−^ mice. (**T**) Spine length in male Cyp46Tg and Tg^−^ mice. (**U**) Spine area frequency distribution histogram of male Cyp46Tg and Tg^−^ mice (two-sample Kolmogorov-Smirnov test, *P* = 0.0480). (**V**) Frequency distribution histogram of spine length from apical collateral dendrites of male Cyp46Tg and Tg^−^ mice (two-sample Kolmogorov-Smirnov test, *P* = 0.0162 respectively). *N* = 5 to 6 mice per group per sex. Data are represented as means ± SEM. *P < 0.05 and ***P < 0.001.

To elucidate whether the observed sex-specific behavioral changes were accompanied by alterations in dendritic spine density and morphology, we performed Golgi staining and examined the apical collateral dendrites (*stratum radiatum*) from CA1 pyramidal neurons of 18-month-old Cyp46Tg mice (Fig. 2, A to L). Female Cyp46Tg mice showed no differences in spine density (number of spines per micrometer; Fig. 2M) but showed a significant increase in both spine area and length compared to their littermates (Fig. 2, N and O), as well as higher frequency distribution of spine area and length (Fig. 2, P and Q). Differently from females, Cyp46Tg males presented significantly lower spine density (Fig. 2R) and lower frequency of spine area and length when compared to controls (Fig. 2, U and V).

To confirm these findings, we measured levels of synaptic proteins in both total hippocampal homogenates and synaptosome enriched fractions from 18-month-old Cyp46Tg and Tg^−^ mice (fig. S2). Transgenic females have higher total hippocampal levels of NMDAR1, p-NMDAR2A, and postsynaptic density 95 (PSD95) than their respective controls (fig. S2, A to C) (*4*), while transgenic males show unchanged levels of NMDAR1 and p-NMDAR2A and decreased levels of PSD95 (fig. S2, E to G). When we analyzed the synaptosome enriched fractions, we observed a significant increase of PSD95 and synaptophysin in Cyp46Tg females (fig. S2, J and K), while transgenic males showed a decrease of both proteins when compared to Tg^−^ mice (fig. S2, N and O).

### Cyp46Tg mice show sex-dependent changes in brain neurosteroid signaling

**Fig. 3.**
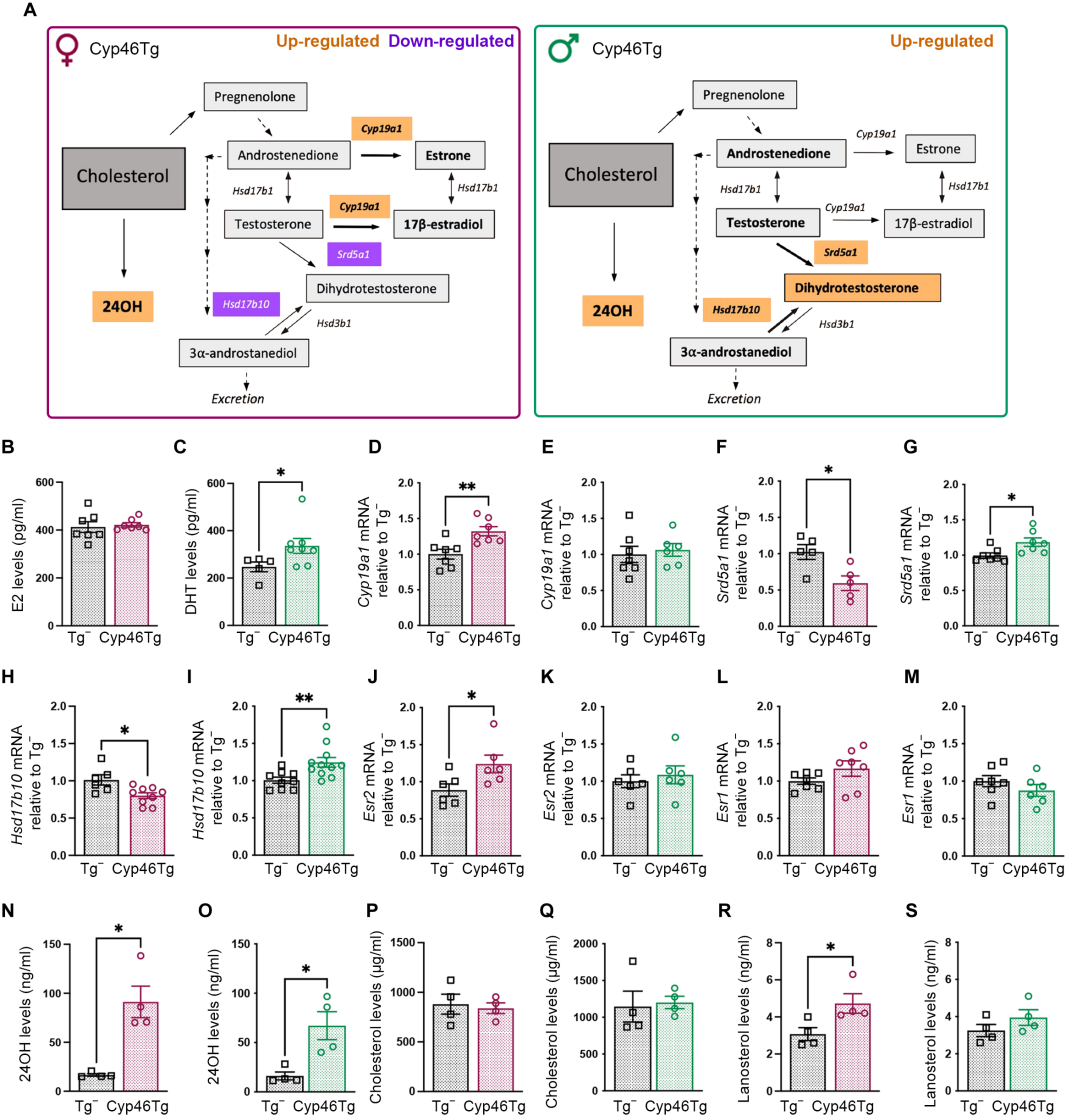
Sex hormone signaling in the hippocampus of 18month-old Cyp46Tg mice. (**A**) Scheme representing the neurosteroid synthesis starting from cholesterol. The genes up-regulated in Cyp46Tg mice are indicated in orange, while the down-regulated ones are indicated in purple. (**B**) Enzyme-linked immunosorbent assay (ELISA) measurements of E2 levels in the hippocampus from female Cyp46Tg and Tg^−^ mice. (**C**) DHT levels from male Cyp46Tg and Tg^−^ hippocampi assessed by ELISA (Mann-Whitney test, *P* = 0.0451). (**D** and **E**) *Cyp19a1* levels in Cyp46Tg females and males in comparison to their Tg^−^ controls (unpaired *t* test, *P* = 0.0053 in females). (**F** and **G**) *Srd5a1* in Cyp46Tg females and males in comparison to their Tg^−^ controls (unpaired *t* test, *P* = 0.00289 and *P* = 0.0193, respectively). (**H** and **I**) *Hsd17b10* in Cyp46Tg females and males in comparison to their Tg^−^ controls (unpaired *t* test, *P* = 0.0134 and *P* = 0.0081, respectively). (**J** and **K**) *Esr2* in Cyp46Tg females and males in comparison to their Tg^−^ controls (unpaired *t* test, *P* = 0.0367 in females). (**L** and **M**) *Esr1* in Cyp46Tg and Tg^−^ females and males. (**N** and **O**) Serum levels of 24OH in female and male Cyp46Tg and Tg^−^ mice (unpaired *t* test, *P* = 0.0036 and *P* = 0.0136, respectively). (**P** and **Q**) Serum cholesterol levels in aged female and male Cyp46Tg and Tg^−^ mice. (**R** and **S**) Brain lanosterol levels in aged female and male Cyp46Tg and Tg^−^ mice (unpaired *t* test, *P* = 0.0395 in females). Gene expression is normalized by *Gapdh*. *N* = 5 to 11 mice per group per sex for RT-qPCR and *N* = 4 mice per group per sex for gas chromatography–mass spectrometry analysis. All data are presented as means ± SEM. *P < 0.05 and **P < 0.01

Sex hormones such as 17β-estradiol (E2) and 5α-dihydrotestosterone (DHT) can be synthesized in the brain, using cholesterol as the main substrate (*39*) (Fig. 3A). We aimed to elucidate whether the sex-dependent differences observed in old Cyp46Tg mice could be mediated by neuroactive steroids, like sex hormones.

We first measured hippocampal levels of E2 and DHT in 18-month-old male and female Cyp46Tg mice by enzyme-linked immunosorbent assay (ELISA). E2 did not change between female groups (Fig. 3B). However, DHT significantly increased in Cyp46Tg males compared to their littermates (Fig. 3C). To determine the mechanisms responsible for this effect, we next assessed the expression levels of key enzymes involved in the biosynthesis of E2 and DHT by RT-qPCR. Aromatase (*Cyp19a1*), responsible for the conversion of testosterone (T) into E2, was significantly increased in female Cyp46Tg mice compared to Tg^−^ both at 9 (fig. S1G) and 18 months of age (Fig. 3D), while no differences were found in male mice (Fig. 3E). 5α-Reductase (*Srd5a1*, converting testosterone to DHT) decreased in 18-month-old transgenic females compared to controls (Fig. 3F), while, in agreement with higher DHT levels, *Srd5a1* levels were increased in 18-month-old Cyp46Tg males (Fig. 3G). Hydroxy-steroid dehydrogenase-17β-10 (*Hsd17b10*, synthesizing DHT from 3α-androstanediol) was decreased in females (Fig. 3H), while it increased in males (Fig. 3I). No changes in *Srd5a1* and *Hsd17b10* were found in 9-month-old female and male Cyp46Tg mice (fig. S1, H to L).

We next determined potential differences in the expression levels of estrogen receptors that could further contribute to the sex-dependent differences observed in aged Cyp46Tg mice. *Esr2* (codifying for estrogen receptor β) was significantly increased only in 18-month-old female Cyp46Tg mice compared to Tg^−^ (Fig. 3J) and not in males. Estrogen receptor β (ERβ) protein levels were higher in the hippocampus of 18-month-old Cyp46Tg females (fig. S3, A and B). We did not find differences in *Esr1* [codifying for estrogen receptor α (ERα)] in female or male Cyp46Tg mice compared to controls (Fig. 3, L and M).

To discard the possibility that sex-dependent outcomes in Cyp46Tg mice are driven by differences in 24OH levels during aging, we measured 24OH, cholesterol, and cholesterol precursor levels in serum and brain of 18-month-old male and female Cyp46Tg and Tg^−^ mice by gas chromatography–mass spectrometry. As expected, Cyp46Tg mice displayed significantly elevated 24OH levels in serum independently of sex and age (Fig. 3, N and O), while serum cholesterol levels remained unchanged (Fig. 3, P and Q). Brain lanosterol levels were higher only in female Cyp46Tg compared to Tg^−^ both at 9 and 18 months of age (Fig. 3, R and S, and fig. S1, R and U). This is in accordance with our previous study, where lanosterol levels were found to increase in young transgenic females (*4*). Brain cholesterol and desmosterol levels remained unchanged in 9-month-old Cyp46Tg mice (fig. S1, Q, T, S, and V).

### 24OH activates neurosteroid signaling in neurons and DHT counteracts its effects

**Fig. 4.**
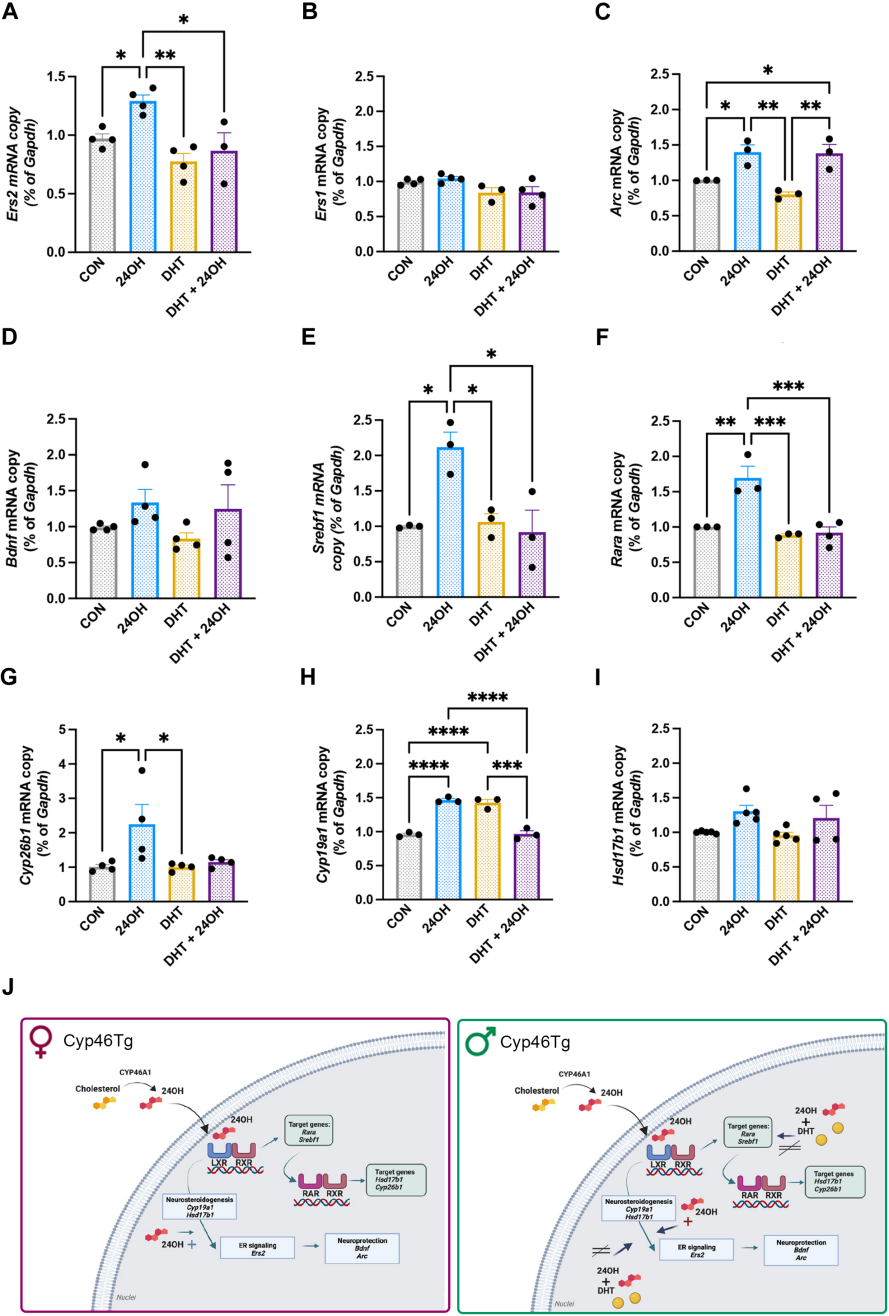
24OH, DHT, and DHT + 24OH treatments in primary neurons. (**A** to **I**) Relative expression levels of different genes normalized by *Gapdh* in hippocampal neurons treated with 24OH, DHT, DHT + 24OH, or vehicle (CON): (A) *Esr2* levels (one-way ANOVA *P* = 0.0030 followed by Tukey’s multiple comparisons test: CON versus 24OH *P* = 0.0497, 24OH versus DHT *P* = 0.0025, 24OH versus DHT + 24OH *P* = 0.0161). (B) *Ers1* levels (C) *Arc* levels (one-way ANOVA *P* = 0.0021 followed by Tukey’s multiple comparisons test: CON versus 24OH *P* = 0.0364, CON versus DHT + 24OH *P* = 0.0449, 24OH versus DHT *P* = 0.0040, DHT versus DHT + 24OH *P* = 0.0048). (D) *Bdnf* levels. (E) *Srebf1* levels (one-way ANOVA *P* = 0.0078 followed by Tukey’s multiple comparisons test: CON versus 24OH *P* = 0.0161, 24OH versus DHT *P* = 0.0220, 24OH versus DHT + 24OH *P* = 0.0111). (F) *Rara* levels (one-way ANOVA *P* = 0.0005 followed by Tukey’s multiple comparisons test: CON versus 24OH *P* = 0.0029, 24OH versus DHT *P* = 0.0010, 24OH versus DHT + 24OH *P* = 0.0008). (G) *Cyp26b1* levels (one-way ANOVA *P* = 0.0304 followed by Tukey’s multiple comparisons test: CON versus 24OH *P* = 0.0491, 24OH versus DHT *P* = 0.0476). (H) *Cyp19a1* levels (one-way ANOVA *P* < 0.0001 followed by Tukey’s multiple comparisons test: CON versus 24OH *P* < 0.0001, CON versus DHT +24OH *P* < 0.0001, 24OH versus DHT + 24OH *P* < 0.0001, DHT versus DHT + 24OH *P* = 0.0001). (I) *Hsd17b1* levels (one-way ANOVA *P* = 0.0449). (**J**) Proposed mechanism of action for 24OH in aged female (left) and male (right) Cyp46Tg mice: In neurons, 24OH binds to LXR, activating the RARE promoter and RARA. 24OH induces neurosteroidogenesis via LXR/RARA, enhancing estrogen receptor (ER) signaling to promote neuroprotection in transgenic females. In contrast, the presence of high levels of DHT + 24OH in male mice counteracts 24OH up-regulating effects on LXR target genes (*Srepf1*, *Rara*, and *Cyp19a1*) and *Ers2*. ImageJ was created with Biorender.com. *N* = 3 to 5 independent experiments, where each experiment was performed in duplicates or triplicates. All data are presented as means ± SEM. *P < 0.05, **P < 0.01, ***P < 0.001, and ****P < 0.0001.

We next used in vitro experiments to clarify whether the metabolic product of CYP46A1, 24OH affects estrogen signaling and neurosteroidogenesis. To determine the effects of 24OH alone and its interplay with DHT, we treated hippocampal neurons in primary culture with 1 μM 24OH, 10 nM DHT, or a combination of both (DHT + 24OH). The concentration of 1 μM 24OH was selected on the basis of the concentration range measured in human brains (*7*), while, for DHT, we chose 10 nM based on previous studies (*40*). In agreement with our in vivo data, *Esr2* expression was significantly up-regulated after 6-hour treatment with 24OH, an effect that was not observed in combination with DHT and when 24OH was used in combination with DHT (Fig. 4A). According to this finding, 24OH treatment also led to higher ERβ protein levels, as determined by immunoblotting for cell nuclear fractions (fig. S4, B and C). No differences were observed for *Esr1* and ERα (Fig. 4B and fig. S4, A and C).

To further clarify the effect of 24OH on ER signaling, we determined the levels of different downstream ER target genes, such as activity-regulated cytoskeletal protein (*Arc*) and brain-derived neurotrophic factor (*Bdnf*). These genes are also known as regulators of synaptic plasticity (*41*, *42*). 24OH treatment triggered a significant increase of *Arc* (Fig. 4C) and 24OH in combination with DHT led to increased *Arc* expression compared to vehicle-treated neurons (Fig. 4C). We did not find changes between treatments in *Bdnf* levels (Fig. 4D).

LXR activation induces transcription of sterol regulatory element binding transcription factor 1 (*Srebf1*) and retinoid acid receptor α (*Rara*) with consequence dimerization of RARA-RXR (see Fig. 4J) (*43*, *44*). Previous findings showed that both LXR and retinoic acid receptor (RAR) signaling induce neurosteroidogenesis (*25*, *45*). Since 24OH binds LXR, we next aimed to confirm whether 24OH modulates neurosteroidogenesis via LXR and RARA. We first confirmed that 24OH activates LXR in neurons by showing an increase of *Srebf1* in 24OH-treated neurons (Fig. 4D), this up-regulation was not present upon DHT and DHT +24OH treatments (Fig. 4D). *Rara* was significantly up-regulated in neurons treated with 24OH (Fig. 4E) but this up-regulation was prevented by DHT and DHT + 24OH conditions. These results were confirmed by Western blot where RARΑ protein levels were elevated upon 24OH treatment, and this increase was prevented in DHT + 24OH treatments (fig. S4, D and E). In agreement with the 24OH activation of *Rara*, its target gene *Cyp26b1* was up-regulated only in 24OH-treated neurons (Fig. 4G). Retinoid X receptor γ (*Rxrg*) was not changing in our experiments (fig. S4F). Last, transcript levels of the neurosteroidogenic enzyme *Cyp19a1* were higher in 24OH treated cells compared to controls, while the treatment DHT + 24OH prevented this increase (Fig. 4H). We did not find differences in *Hsd17b1* between treatments (Fig. 4I).

### *CYP46A1* overexpression protects ovariectomized mice from memory loss

To determine whether *CYP46A1* overexpression and high levels of 24OH sustain neurosteroid signaling upon deprivation of peripheral sex hormones (endocrine aging), we performed the surgical removal of gonads (gonadectomy) in male and female wild-type (GDX-Wt) and Cyp46Tg (GDX-Cyp46Tg) mice at 2 to 3 months of age. Regarding the control groups, we first performed sham surgery in a pilot study to test the effects of sham surgery on behavioral outcomes in males and females (fig. S5). Since sham surgery did not affect cognitive outcomes like the % of alternation in Y-maze (fig. S5, A and B), we used non-operated mice (Wt) as control groups for the effect of gonadectomy.

**Fig. 5.**
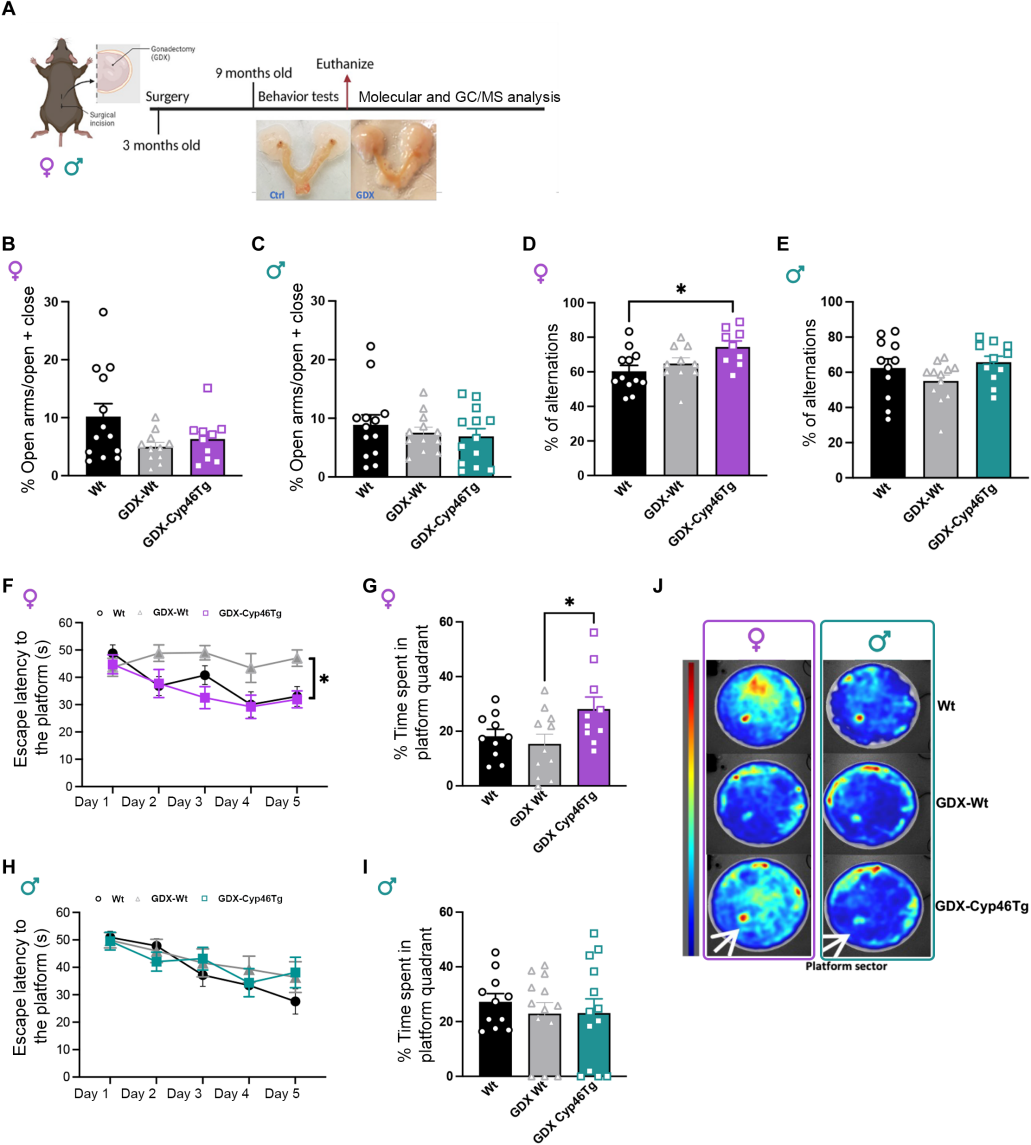
Behavioral tests in gonadectomized mice. (**A**) Experimental design: Female and male Cyp46Tg and wild-type mice underwent gonadectomy at 2 to 3 months of age. At 9 months, the mice were assessed for behavioral tests, after their brains were collected for molecular analysis. The experimental design was created with Biorender.com. (**B** and **C**) Percentage of time spent in open arms during the EPM test in the female and male groups, respectively. (**D**) Percentage of spontaneous alternations in the Y-maze test in female mice (one-way ANOVA *P* = 0.0255 followed by Tukey’s multiple comparisons test: Wt versus GDX-Cyp46Tg *P* = 0.0202). (**E**) Percentage of spontaneous alternations in the Y-maze test in male mice. (**F**) Escape latencies during MWM acquisition phase in the female groups (repeated-measures ANOVA, the effect of days *P* = 0.0018, effect of genotype *P* = 0.0030, followed by Tukey’s multiple comparison test: for day 3 GDX Wt versus GDX Cyp46Tg *P* = 0.0096, for day 5 Wt versus GDX Wt *P* = 0.0217, GDX Wt versus GDX Cyp46Tg *P* = 0.0065). (**G**) Percentage of time spent in the quadrant target during probe test in female mice (one-way ANOVA *P* = 0.0448, followed by Tukey’s multiple comparisons test: GDX-Wt versus GDX-Cyp46Tg *P* = 0.0441). (**H**) MWM acquisition phase expressed as escape latency in the male mice groups (repeated-measures ANOVA, the effect of days *P* < 0.0001). (**I**) Percentage of time spent in the quadrant target during the probe test in male mice. (**J**) Heatmaps from the MWM probe test in female and male mice: The occupancy rate is represented by a color map (in red color the most visited zones). *N* = 9 to 13 mice per group per sex. Following the exclusion criteria, we removed four males and four females from the analyses of % alternation in the Y-maze and four females and two males from the MWM probe test analyses. All data are presented as means ± SEM. *P < 0.05.

Six months after surgery, all groups of mice underwent behavioral tests (Fig. 5A). No differences were found in the EPM test between groups, for males or females (Fig. 5, B and C). In the Y-maze test, GDX-Cyp46Tg females displayed a higher percentage of correct alternations when compared to Wt (Fig. 5D), while we did not observe differences between the male groups (Fig. 5E). In the MWM test, female GDX-Wt mice did not learn to locate the platform and showed higher escape latency times (time spent to reach the hidden platform) than both Wt and GDX-Cyp46Tg (Fig. 5F). During the probe test, GDX-Cyp46Tg spent more time than GDX-Wt in the target quadrant (Fig. 5G). Differently from female mice, gonadectomy did not affect memory performance in male Wt and Cyp46Tg mice assessed in the MWM test, during the learning and probe test (Fig. 5, H and I). The representative heatmaps support the results from the MWM probe test (Fig. 5J). All groups of mice did not differ in exploratory activity (number of entries) in the Y-maze and swim speed in the MWM (fig. S5, C to F), suggesting that gonadectomy did not alter functional outcomes known to affect the test performances.

### Gonadectomy alters CYP46A1 and neurosteroid signaling in a sex-dependent manner

**Fig. 6.**
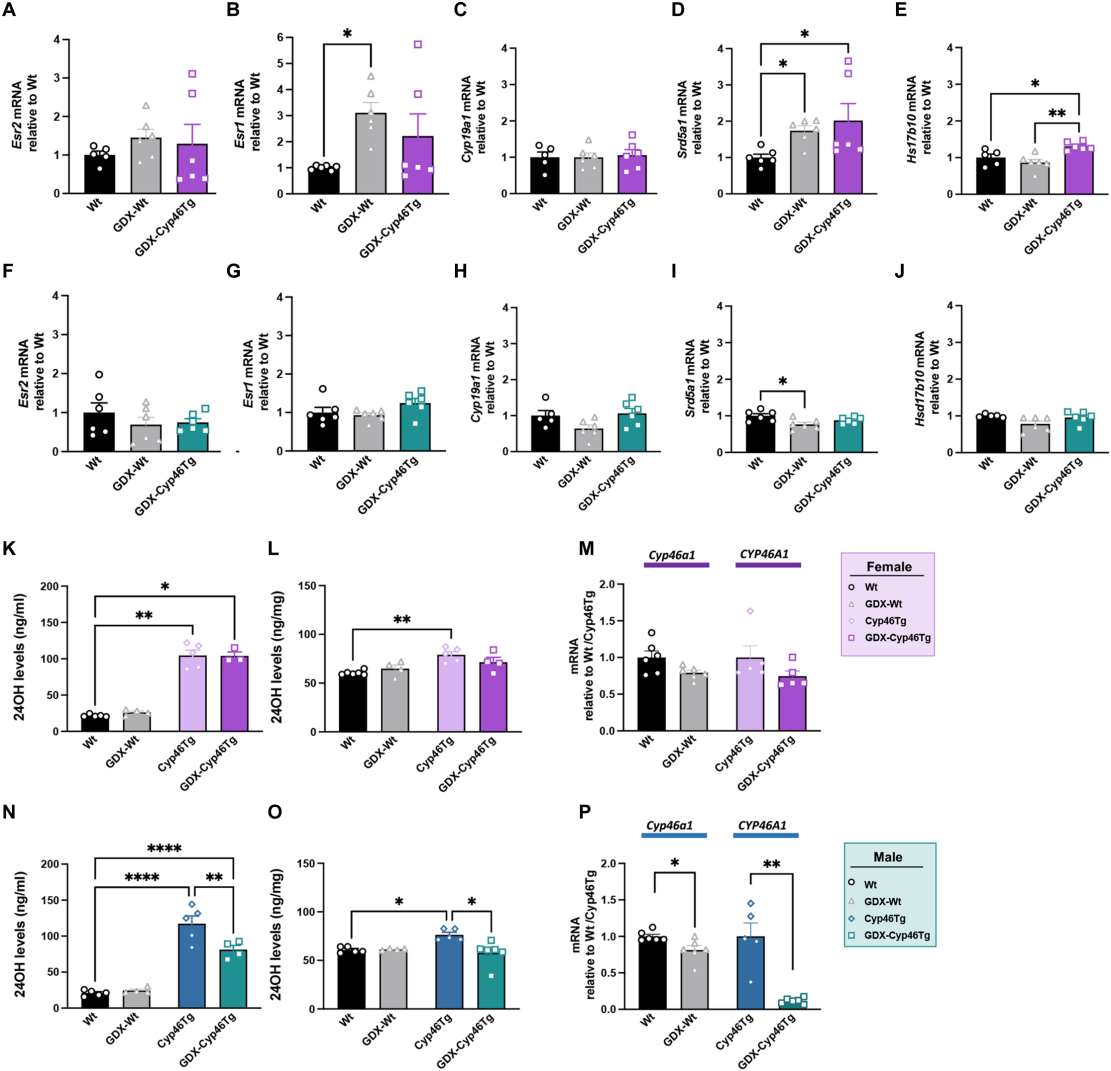
Neurosteroid signaling, 24OH, and *CYP46A1* levels in gonadectomized mice. (**A**) Hippocampal levels of *Esr2* in female GDX-Cyp46Tg, GDX-Wt, and Wt mice. (**B**) Hippocampal levels of *Esr1* in female mice (Kruskal-Wallis test, *P* = 0.0208, followed by Dunn’s multiple comparisons test: Wt versus GDX-Wt *P* = 0.0242). (**C**) Hippocampal levels of *Cyp19a1* in female mice. (**D**) Hippocampal levels of *Srd5a1* in female mice (Kruskal-Wallis test *P* = 0.0275, followed by Dunn’s multiple comparisons test: Wt versus GDX-Wt *P* = 0.0283). (**E**) Hippocampal levels of *Hsd17b10* in female mice (one-way ANOVA *P* = 0.0032, followed by Tukey’s multiple comparison test: Wt versus GDX-Cyp46Tg *P* = 0.00393 and Wt-GDX versus GDX-Cyp46Tg *P* = 0.0027). (**F**) Hippocampal levels of *Esr2* in male mice. (**G**) Hippocampal levels of *Esr1* in male mice. (**H**) Hippocampal levels of male *Cyp19a1* mice. (**I**) Hippocampal levels of *Srd5a1* in male mice. (**J**) Hippocampal levels of *Hsd17b10* in male mice (one-way ANOVA *P* = 0.0212, followed by Tukey’s multiple comparisons test Wt versus GDX-Wt *P* = 0.0163). (**K**) 24OH levels in serum from female GDX-Cyp46Tg, GDX-Wt, Cyp46Tg, and Wt mice. (**L**) 24OH brain levels from female mice. (**M**) *Cyp46a1* levels in female GDX-Wt and Wt mice and *CYP46A1* levels in GDX-Cyp46Tg mice compared to age-matched Cyp46Tg females. (**N**) Serum 24OH levels from male mice (one-way ANOVA *P* = 0.0043). (**O**) Brain 24OH levels in male mice (One-way ANOVA *P* = 0.0024). (**P**) *Cyp46a1* levels in male GDX-Wt and Wt mice and *CYP46A1* levels in male GDX-Cyp46Tg mice compared to age-matched Cyp46Tg mice (unpaired *t* test *P* = 0.0212 and *P* = 0.0005 respectively). *N* = 4 to 6 mice per group per sex. All data are presented as means ± SEM. *P < 0.05, **P < 0.01, and ****P < 0.0001.

To investigate the effects of gonadectomy on brain sex-hormone signaling, we measured mRNA levels of estrogen receptors and neurosteroidogenic enzymes in the hippocampus of Cyp46Tg and Tg^−^ mice (Fig. 6). Regarding females, while the expression levels of *Esr2* did not change among groups (Fig. 6A), *Esr1* was significantly up-regulated in GDX-Wt compared to Wt animals (Fig. 6B). *Cyp19a1* mRNA levels were not changed between groups (Fig. 6C); however, female GDX-Wt and GDX-Cyp46Tg mice showed a substantial increase of *Srd5a1* compared to Wt mice (Fig. 6D). Last, *Hsd17b10* expression was significantly increased in GDX-Cyp46Tg females compared to GDX-Wt and Wt (Fig. 6E). No significant effects of gonadectomy on *Ers2*, *Ers1*, *Cyp19a1*, and *Hsd17b10* were observed in males (Fig. 6, F to J). These mice showed a decrease of *Srd5a1* levels that was prevented in GDX-Cyp46Tg (Fig. 6I).

We next assessed whether gonadectomy affects *CYP46A1* and 24OH levels. In GDX-Cyp46Tg females, 24OH levels were unchanged in both serum and brain compared to Cyp46Tg used as controls (Fig. 6, K and L). Cyp46Tg females showed similar levels of human *CYP46A1* regardless of ovariectomy (Fig. 6M), and GDX-Wt females showed substantially lower mouse *Cyp46a1* levels than Wt females (Fig. 6M). In contrast, GDX-Cyp46Tg males showed significantly reduced 24OH levels compared to Cyp46Tg males both in serum (Fig. 6N) and the brain (Fig. 6O). In agreement with these results, *CYP46A1* was markedly reduced upon gonadectomy in Cyp46Tg males (Fig. 6P).

### CSF 24OH is negatively associated with total tau in women in an AD clinical cohort

We lastly investigated the association between 24OH and biomarkers of AD pathology and neurodegeneration in CSF samples from male and female patients with SCI, MCI, and AD. Both men and women had a similar age distribution, similar cognition levels as measured by the Mini Mental State Examination (MMSE) score, and an equal representation of the diagnostic groups (Table 1). Levels of Aβ42, total tau (t-tau), phosphorylated tau 181(p-tau), p-tau/Aβ42 ratio, neurofilament light chain (NFL), and 24OH were equally distributed across men and women (Table 1). Levels of 24OH did not correlate with age (*P* = 0.951) or show sex-dependent differences (fig. S6F). To investigate the association between 24OH and different biomarkers for AD and neurodegeneration, we performed linear regression models for Aβ42, t-tau, p-tau, p-tau/Aβ42, and NFL, adjusting for age and diagnosis (Table 2). Higher 24OH levels were associated with decreased p-tau, p-tau/Aβ42, and NFL in the total cohort (Table 2). No correlation was found between 24OH and Aβ42. To test a sex-specific effect of 24OH, we examined the interaction between 24OH and sex on Aβ42, t-tau, p-tau, p-tau/Aβ42, and NFL. As shown in Table 2, we found a significant interaction between 24OH and sex for the association with t-tau (Table 2). When we performed stratified analysis to analyze each sex separately, we observed that the associations between 24OH and t-tau, p-tau, p-tau/Aβ42, and NFL were seen only in women and not in men (Table 2 and fig. S6, A to E).

**Table 1.** Demographic, clinical, and biomarker data. Demographic factors, clinical characteristics, and CSF biomarkers were compared using χ^2^ and Mann-Whitney *U* tests. Continuous data are shown as means (± SD). NFL, neurofilament light chain.

Variables	Men (*n* = 42)	Women (*n* = 48)	*P* value
**Demographics**			
Age	65.0 (± 6.5)	65.8(± 7.7)	0.537
MMSE score	25.7 (± 5.2)	27.1(± 2.8)	0.403
**Diagnosis**			
SCI	12	18	0.669
MCI	15	15	
AD	15	15	
**CSF biomarkers**			
Aβ42, pg/ml	683.3 (± 228.4)	722.8 (± 269.8)	0.450
Aβ42 positive % (<550 pg/ml)	33.3	29.2	0.670
t-tau, pg/ml	397.2 (± 196.5)	444.1 (244.3)	0.462
p-tau, pg/ml	67.7 (± 27)	68.2 (± 22.7)	0.442
p-tau/Aβ42	0.11 (± 0.05)	0.11 (± 0.06)	0.796
NFL, pg/ml	1387.6 (± 674.7)	1330.8 (± 850.1)	0.290
24OH, pg/ml	2206.0 (± 1194.9)	2124.0 (± 980.0)	0.913

**Table 2.** Associations between CSF 24OH and biomarkers of AD pathology and neurodegeneration. Associations of Aβ42, t-tau, p-tau, p-tau/Aβ42, and NFL with 24OH were examined using multiple linear regression models, adjusting for age and diagnosis. For the model testing the interaction effect of 24OH with sex in the whole cohort, the interaction term 24OH × sex was additionally included and the reported β coefficients were calculated using mean-centered 24OH levels. Multiple linear regression models, adjusted for age and diagnoses, were tested also in men and women separately. Data shown are β coefficients with *P* values (significant *P* values are marked in bold).

	Aβ42 β (*P* value)	t-tau β (*P* value)	p-tau β (*P* value)	p-tau/Aβ42 β (*P* value)	NFL β (*P* value)
**All participants**					
24OH	0.030 (0.628)	−0.85 (0.324)	−0.212 (**0.041**)	−0.158 (**0.043**)	−0.256 (**0.001**)
24OH × sex	0.076 (0.371)	−0.304 (**0.008**)	−0.195 (0.166)	−0.176 (0.097)	−0.095 (0.367)
**Men**					
24OH	−0.043 (0.670)	0.121 (0.365)	−0.028 (0.865)	−0.053 (0.669)	−0.217 (0.082)
**Women**					
24OH	0.060 (0.712)	−0.235 (**0.033**)	−0.294 (**0.021**)	−0.223 (**0.009**)	−0.230 (**0.026**)

## DISCUSSION

In this study, we investigated the sex-specific effects of CYP46A1 activation on cognition in aged mice and its relation with AD biomarkers. During physiological aging, mice show reduced spontaneous alternations in the Y-maze and worsened spatial learning (*46*), together with a decrease in dendritic spine density and total number of spines in cortical and hippocampal regions (*47*, *48*). These morphological alterations are also observed in AD mouse models and patients (*48*–*50*). Here, we show that *CYP46A1* overexpression in aged female mice enhances spatial memory and dendritic spine length and area, protecting against the deleterious effects of aging. On the contrary, overexpression of *CYP46A1* in aged males triggers a decline in spatial-dependent memory retention and decreased hippocampal spine density. In addition, these mice show anxiety-like behavior, which has been previously linked to decreased spine density in CA1 regions (*51*). Our results are in agreement with other studies reporting beneficial effects of *CYP46A1* up-regulation in memory performances during aging and AD (*4*, *16*–*18*) in female mice; nevertheless, these studies were not performed in males. Chronic treatments with low doses of efavirenz, a CYP46A1 activator, ameliorated cognition in the MWM test and increased synaptic proteins in a mouse model of early amyloidogenesis (*19*). The authors do not report sex differences in behavior, and, to our knowledge, sex was not taken into account when analyzing levels of brain synaptic proteins.

We observe divergent effects between sexes at 18 months but not at 9 months of age, suggesting that effects of *CYP46A1* overexpression develop with age, conferring protection in females and contributing to further deterioration in males. Differences in the levels of 24OH, the metabolic product of CYP46A1, cannot account for this effect, as our data show that transgenic males and females have similar amounts of 24OH in serum and brain, and its levels are preserved throughout aging. This suggests that, even if we used mixed cohorts of heterozygous and homozygous mice, the observed outcomes are not affected by a gene dose effect of *CYP46A1*. Nonetheless, in future studies, it may be recommendable to define zygosity.

Considering that 24OH interacts with LXR, and its role in promoting neurosteroidogenesis (*22*, *25*), we explored whether the differences seen in our model could be explained by differential regulation of neurosteroid synthesis between males and females. In this context, a previous study conducted on Cyp46Tg mice at a young age did not show changes in brain levels of LXR target genes (*52*). This seems in contrast with our results, where LXR downstream signaling is altered, however, mainly in old age. In this study, Cyp46Tg males showed an up-regulation of both *Hsd17b10 and Srd5a1* that was consequently accompanied by increased levels of DHT. According to previous findings, high DHT concentrations could contribute to anxiety-like behavior and reduction of CA1 spine density, worsening memory performances (*53*–*55*). This could explain the phenotype seen in Cyp46Tg males. However, DHT has also been described as neuroprotective (*40*, *56*). We show that neurons treated with 24OH have increased *Esr2*, nuclear levels of ERβ, E2 downstream targets like *Arc* (*41*, *42*), and RARA signaling and that most of these effects are absent when 24OH treatment is performed in the presence of DHT. We still observe an increase in *Arc* after a combination of 24OH and DHT treatments, which could be explained by limitations of our experimental setup (concentrations, time, etc.) or by other unknown mechanisms contributing to the blockage of 24OH effects mediated by DHT. An additional limitation of this study is that we have not measured levels of glucocorticosteroids, known to affect anxiety, memory, and neurodegeneration (*57*).

Together, our data suggest that the positive effects observed in Cyp46Tg females are a consequence of sustained activation of brain estrogen signaling by 24OH during aging. Differently from males, Cyp46Tg females showed higher levels of ERβ and up-regulation of *Esr2*, *Cyp19a1*, and *Hsd17b1*, which would lead to increased E2 levels (*58*). E2 is highly lipophilic and can cross the BBB from the periphery into the brain. Aging female mice do not naturally model human menopause. Nevertheless, females over 12 to 13 months show a reduction of oocyte follicles and a consequent decrease in blood E2 levels (*59*) that could affect brain E2 concentration, thereby compromising E2-dependent neuroprotective mechanisms (*60*). We suggest that *CYP46A1* overexpression contributes to stabilizing E2 levels in the brain upon endocrine aging. In support of this hypothesis, our data show that E2 hippocampal levels are more homogenous among aged Cyp46Tg females compared to female controls, and it is well known that E2 potentiates hippocampal plasticity, as well as the abundance and density of dendritic spines (*61*–*65*).

To further determine the impact of *CYP46A1* overexpression in conditions that better represent the decline in E2 levels experienced by women during menopause or following oophorectomy in premenopausal age, we performed long-term ovariectomy. Gonadectomy in mice causes memory impairments, especially in females, that can be reverted by exogenous E2 supplementation (*66*–*68*). In our study, ovariectomy led to cognitive deficits in female Wt mice, especially in the MWM test, that were significantly mitigated by *CYP46A1* overexpression. In males, gonadectomy did not have major negative effects on memory and on the expression levels of neurosteroid enzymes. Unlike females, Cyp46Tg males showed a decrease of 24OH levels in serum and brain that did not affect cognition. It should be noted that there was a marked down-regulation of the human transgene in GDX Cyp46Tg males, suggesting the presence of a DHT/T-dependent regulation of CYP46A1 expression.

Here, we used a mouse model of global *CYP46A1* overexpression, which led to high levels of 24OH in the brain and serum (*52*). While, in this model, the *CYP46A1* transgene is mostly detected in the brain, and specifically restricted to neurons (*52*), we cannot exclude the presence of peripheral sources of 24OH possibly contributing to the phenotypes observed in this study.

To determine the relevance of our findings for neurodegenerative diseases like AD, we conducted an exploratory study and analyzed CSF from patients in a memory clinic cohort. As AD-related comorbidities like hypercholesterolemia, diabetes, and hypertension affect cholesterol metabolism (*69*), we excluded individuals with these AD risk factors. In this cohort, 24OH levels did not differ between AD, SCI, and MCI, as reported in other studies (*8**,* *70**,* *71*). We observed a sex-specific effect of 24OH on t-tau, a known marker of neurodegeneration. Our data show that higher 24OH is associated with lower t-tau only in women, suggesting a sex-specific protective effect of 24OH. This finding supports the idea that the sex-specific effects we observe in mice may be translatable to women. However, we acknowledge that our sample size is limited and the effects observed are weak to moderate, warranting further investigations in larger cohorts to strengthen our conclusions.

In summary, our findings show that *CYP46A1* overexpression and consequently, elevated 24OH, modulate neuroactive steroid signaling in the brain in a sex-dependent manner, leading to neuroprotective effects only in females during both chronological and endocrine aging. Our current work shows that gonadectomy leads to decreased *Cyp46a1* expression in mice. Furthermore, a recent study has shown higher CYP46A1 levels in young, cognitively normal women in comparison to men, suggestive of a role for sex hormones in CYP46A1 regulation (*72*). Together, these findings encourage further investigation of the interplay between sex hormone levels, brain cholesterol turnover, and neurodegeneration in humans. This is particularly important from a clinical perspective, considering that early menopause represents a female-specific risk factor for cognitive decline and exacerbates neuropathology in AD (*30*–*32*). In this scenario, CYP46A1 activators may become valuable modulators of neurosteroidogenesis, specifically by enhancing brain E2 signaling in women at risk for AD. Our data also suggest that targeting CYP46A1 as a therapy for AD or other neurodegenerative diseases may lead to different outcomes in men and women, and this should be considered when designing therapeutic strategies. Last, this study highlights and confirms the importance of considering the sex dimension in both preclinical and clinical studies of neurodegenerative disorders.

## MATERIALS AND METHODS

### Transgenic mice

We used mixed colonies of heterozygous and homozygous female and male *CYP46A1* HA-tagged transgenic mice (Cyp46Tg) (*52*) at 9 and 18 months of age. As controls, we used sex- and aged-matched Wt littermates (Tg^−^). The mice were kept under controlled temperature (21 ± 1°C) and humidity (55 ± 5%) on a 12-hour light-dark cycle and food and water were provided ad libitum. All experimental procedures on mice were performed following the local national animal care and use guidelines of Sweden and approved by the Swedish Board of Agriculture (ethical permit ID 4884/2019 and 2199/2021). All possible efforts were made to minimize the suffering and distress of the animals.

### Rat primary culture and treatments

Hippocampal tissue was isolated from E18 Sprague-Dawley rat embryos (Charles River Laboratories). Neurons in primary culture were seeded (12,5000 cells/cm^2^) in neurobasal media with 2% B-27, 2 mM GlutaMAX, penicillin (100 U/ml), streptomycin (100 μg/ml) (Thermo Fisher Scientific) and incubated at 37°C in a humidified 5% CO_2_-containing atmosphere. All experiments were performed after 10 days in culture. For treatments, the medium was removed and replaced with neurobasal media with 1 μM of 100% 24OH (Instruchemie, Netherlands) prepared in ethanol (EtOH) and 10 nM DHT (Sigma-Aldrich) resuspended in methanol (MeOH). Medium with the same final concentration of vehicles EtOH/MeOH was used as a control.

### Behavioral tests

We assessed the mice in EPM, Y-maze, and MWM tests. The order of tests was chosen on the basis of the stress level associated with the procedure (*73*). Mice were placed in the experimental room for 30 min for habituation; all tests were run between 9:00 and 15:00 in white light and by the same experimenters. EPM and Y-Maze were performed as previously described (*74*). For the analyses of correct alternation percentage in the Y-maze test, we included only those mice that performed above five entries. For the MWM test, the mice were trained to find a hidden platform as reported by (*4*, *70*). Mice performing thigmotaxis for the whole trial duration (60 s) and mice floating over 15 s were removed from the analyses of the learning phase and probe test. Data were collected using the video-tracking system Ethovision XT-17 (Noldus, Netherlands), connected to a video camera placed above the equipment.

### Golgi staining and dendritic spine analysis

After behavior, we randomly selected female and male Cyp46Tg and Tg^−^ mice and processed them for Golgi analysis. We stained 150-μm-thick coronal sections from the left brain hemispheres with the Golgi-Cox solution using the FD Rapid GolgiStain Kit (NeuroTechnologies, USA), following the manufacturer’s instructions.

We acquired images from a single focal plane and a merge z-stack with a light microscope (Nikon Eclipse E800) with 100× objective, oil immersion (NA: 1.30). We performed the analysis on apical collateral dendrites (stratum radiatum) from CA1 pyramidal neurons. For the analyses, we selected neurons based on the following criteria: (i) neurons relatively complete (two orders or greater of dendrites were entirely visible and in focus); (ii) neurons fully impregnated with staining; and (iii) minimal or no overlap with other labeled neurons. We have selected 5 to 10 different neurons per mouse and we measured up to one to two dendrites per neuron for a total dendritic length of at least 300 μm per animal. All analyses were conducted blinded to genotype. Imaging analyses were performed with ImageJ software (National Institutes of Health, USA).

### Gonadectomy

Three-month-old male and female mice were gonadectomized bilaterally to remove endogenous gonadal hormones. Mice were anesthetized with a mixture of 0.25 mg of midazolam, 0.025 mg of medetomidine, and 12 μg of fentanyl. Male mice were stitched with resorbable sutures. In female mice, the incisions were closed using clips and removed after 2 weeks. Mice received postsurgical injection of 10 μg of flumazenil and 0.05 mg of atipamezole to wake up, and an injection of 5 μg of buprenorphine to ease any potential pain. The mice were kept warm during and after the surgery.

### Western blotting

Brain tissue and neurons were lysed in radioimmunoprecipitation assay (RIPA) buffer (50 mM tris, 150 mM NaCl, 1% Triton X-100, and 0.1% SDS) and protease and phosphatase inhibitor cocktail (1:100; Sigma-Aldrich). Western blot was performed as previously described (*74*). The primary antibodies stated as follows: anti-RARα (1:500; Abcam, ab28767), anti-ERα (1:1000; Abcam, ab3575), anti-ERβ (1:1000; Santa Cruz Biotechnology, sc-53494), Lamin A/C (1:1000; Sigma-Aldrich, SAB4200236), anti-NMDA receptor 2A (1:250; Abcam, UK), anti-NMDA receptor 1 (1:1000; BD Biosciences, UK), anti-PSD95 (1:1000; Cell Signaling Technology), anti-synapsin (1:1000; Abcam, ab64581), anti-synaptophysin (1:1000; Cell Signaling Technology, #4329), and tubulin (1:10,000; Sigma-Aldrich, T9026). Secondary antibodies anti-rabbit, anti-mouse, and anti-goat IgG were used at 1:10,000 dilution (LI-COR Biosciences, Germany). Immunoreactivity was detected by infrared fluorescence with the LI-COR Odyssey system and quantified with ImageJ software by densitometry analysis of immunoreactive bands.

### Extraction of crude synaptosomal fractions

We mechanically homogenized brains from 18-month-old Cyp46Tg and their littermate control mice in cold lysis buffer [1.5 M sucrose, 100 mM Hepes, and 10 ml Milli-Q water supplemented with phosphatase inhibitor cocktail and protease inhibitor 1:100 (pH 7.5)]. The ratio of lysis buffer was 1 ml per 100 mg of tissue. The homogenates were centrifuged at 1000*g* for 10 min at 4°C and the supernatant was transferred to a new 2-ml reaction tube and the pellet was discarded (P1). The resulting supernatant was centrifugated at 12,000*g* at 4°C for 20 min. The supernatant fraction (S2) was immediately frozen, and the pellet (P2) composed of crude synaptosomes resuspended in 75 μl of RIPA buffer. The S2 and P2 fractions were processed for Western blotting.

### Nuclear fractionation

We used the NE-PER kit (Pierce, USA) to isolate the nuclear and cytosolic fractions from hippocampal cultured neurons. The procedure was performed following the manufacturer’s protocol. A protease inhibitor cocktail (1:500; Sigma-Aldrich) was added freshly. We evaluated the purity of the fractions immunoblotting with antibody lamin A/C (1:1000; Sigma-Aldrich).

### RNA extraction and real-time PCR (RT-qPCR)

Total RNA from primary neurons was isolated as previously described (*75*). Mouse hippocampi were homogenized in the RLT buffer from RNeasy Mini Kit (Qiagen, 74106) with a tissue grinder gun and further processed according to the manufacture protocol for RNA extraction. Retro transcription was done using a High-Capacity cDNA Reverse Transcription Kit (Applied Biosystems, 4368814). mRNA copy numbers relative to *Gapdh* mRNA levels were measured by real-time PCR using Taqman Universal MasterMix (Applied Biosystems, USA) and the following primers: *Esr1*, *Esr2*, *Arc*, *Bdnf*, *Cyp19a1*, *Sdr5a1*, *Hsd17b10*, *Hsd17b1*, *Rara*, *Cyp26b1*, *Srepb1*, *Rxrg*, and *Gapdh* (Applied Biosystems, USA).

### Enzyme-linked immunosorbent assay

ELISA for quantitative analysis of DHT and E2 (Lbio and Abcam, respectively) in mouse brain tissue was adapted to the manufacturer’s instruction. Twenty milligrams of dissected hippocampus were homogenized in lysis buffer [tris-HCl 50 mM, NaCl 150 mM, and 1% Triton X-100 (pH 7.4)]. MeOH was added for the extraction of steroidal hormones. After incubation and centrifugation, the supernatant was transferred to a new tube, dried, and resuspended in the ELISA-compatible buffer solution.

### Mass spectrometry analysis

Eighteen- and 9-month-old mice were euthanized, and their trunk blood and brain tissue were subsequently extracted and processed to analyze 24OH and cholesterol using isotope dilution mass spectrometry. The analysis of cholesterol and its precursors was conducted by combined gas chromatography with mass spectrometry, as previously described (*4*).

### Memory clinic patient population

The study included 90 patients (43 men and 48 women) diagnosed with SCI, MCI, or AD. The population involved participants who were not using medications for diabetes, hypercholesterolemia, and hypertension. This cohort is described in (*76*) and the key demographics of the diagnostics groups are shown in table S1. The patients were examined at Karolinska University Hospital memory clinic, Sweden. Patients with MCI were diagnosed using the consensus criteria for MCI (the patients show both subjective and objective cognitive impairments of one or more cognitive domains, without impairment of daily activities and dementia) (*77*). Objective cognitive impairment was defined as a test performance of 1.5 SD below what is expected on the basis of age and education. The diagnosis of dementia was made according to the Diagnostic and Statistical Manual of Mental Disorders criteria, and AD etiology was diagnosed using the National Institute of Neurological and Communicative Disorders and Stroke–Alzheimer’s Disease and Related Disorders Association criteria by McKhann *et al.* (*78*). Patients who did not meet the criteria for MCI or dementia were considered patients with SCI.

The study was approved by the Regional Ethical Review Board in Stockholm, and written informed consent was obtained from all patients. CSF samples from memory clinic patients were from the biobank and database GEDOC. All participants had written informed consent and permissions permission from the Swedish Ethical Review Authority to conduct the research has been admitted (2019-06056).

### CSF biomarkers

CSF samples were collected by standard lumbar puncture between the L3/L4 or L4/L5 intervertebral space using a 25-gauge needle. Aβ42, t-tau, and p-tau 181 concentrations were measured on fresh samples by ELISA (Innogenetics, Belgium), according to standardized protocols in the clinic. 24OH was analyzed as the sum of the esterified and free molecule by liquid chromatography and mass spectrometry as previously described (*79*) but with the addition of a base hydrolysis step in 0.35 M KOH before solid phase separation of total oxysterols from cholesterol. NFL was measured as reported previously (*80*).

### Statistical analysis

The data are expressed as means ± SEM, with *N* indicating the number of mice or technical experiments performed. When comparing two groups, the *t* test or Mann-Whitney test was applied depending on data distribution. One-way and two-way analyses of variance (ANOVAs) followed by Tukey’s multiple comparisons test were used to analyze data with two or more independent variables. For non-normally distributed data, we used the Kruskal-Wallis test, followed by Dunn’s multiple comparisons test. Repeated-measures ANOVA was used for the MWM data. Memory clinic population data were compared across men and women in the cohort with Mann-Whitney *U* tests for continuous variables and χ^2^ for categorical. Correlations were performed by Spearman analysis. Aβ42, p-tau, p-tau/Aβ42, NFL, and 24OH were log-transformed and t-tau was square root–transformed to increase normality. We performed separate multiple linear regression models in pairwise combinations between 24OH and each of the biomarkers (Aβ42, t-tau, p-tau, p-tau/Aβ42, and NFL) in all participants and in men and women separately, where 24OH was set as the independent variable in all models. For the interaction models in the total cohort, the interaction term 24OH × sex was added. 24OH was mean-centered to facilitate for the interpretation of the coefficients in the interaction analysis. All models were adjusted for age and diagnosis. In an additional linear regression model, we adjusted for age, diagnoses, and ApoE in 53 participants. GraphPad Prism 8 software and IBM SPSS 27 (SPSS Inc., Chicago) were used and *P* < 0.05 was considered significant.
