# Supplementary material for: CYP46A1-mediated cholesterol turnover induces sex-specific changes in cognition and counteracts memory loss in ovariectomized mice
Source: Sci Adv. 2024 Jan 24;10(4):eadj1354. doi: 10.1126/sciadv.adj1354 (PMC10807813; doi:10.1126/sciadv.adj1354)
Supplement: Supplementary file 1 — Figs. S1 to S6 Tables S1 and S2 [file sciadv.adj1354_sm.pdf]

Science Advances
AAAS

Supplementary Materials for

CYP46A1-mediated cholesterol turnover induces sex-specific changes in cognition and counteracts memory loss in ovariectomized mice

María Latorre-Leal *et al.*

Corresponding author: Silvia Maioli, silvia.maioli@ki.se

Sci. Adv. **10**, eadj1354 (2024)
DOI: 10.1126/sciadv.adj1354

This PDF file includes:

Figs. S1 to S6
Tables S1 and S2

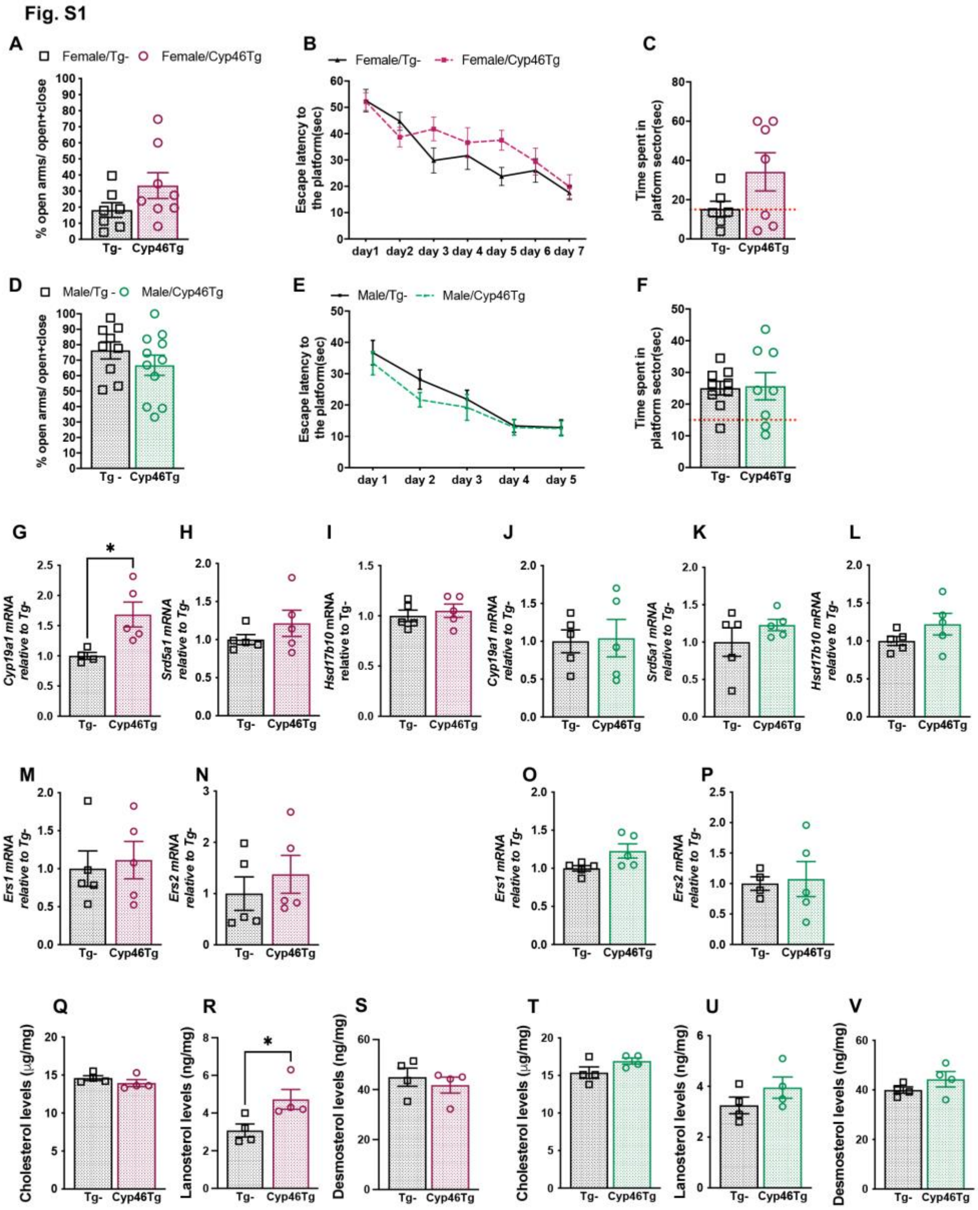

Fig S1. Behavioral tests and molecular analyses in adult Cyp46Tg mice. A) Percentage of time spent in open arms in the EPM test performed by 9 months old Cyp46 Tg female mice compared to Tg- mice **B)** Escape latency over 5 days acquisition phase in MWM test in 9Cyp46 Tg and Tg- female mice **C)** Time spent in the quadrant where the platform was located during the MWM probe test in Cyp46Tg and Tg- female mice. **D)** EPM test performed in 9 months old Cyp46 Tg male mice compared to Tg- mice expressed as percentage of time spent in open arms. **E)** MWM acquisition phase in Cyp46 Tg male mice compared to Tg- male mice over 5 days. **F)** MWM probe test in Cyp46Tg and Tg- male mice. **G-L)** Hippocampal expression levels of *Cyp19a1, srd5a1, hsd1b10* in Cyp46Tg and Tg- female and male mice (unpaired t test, P=0.0401, for *Cyp19a1* in females).**M-P)** Hippocampal expression levels of *Ers1 and Ers2* in Cyp46Tg and Tg- female and male mice. **Q-V)** Serum cholesterol and brain lanosterol and desmosterol levels in Cyp46Tg and Tg- female and male mice Data are shown as mean ± SEM. N=11/4 mice group/sex. * P< 0.01

Fig. S2

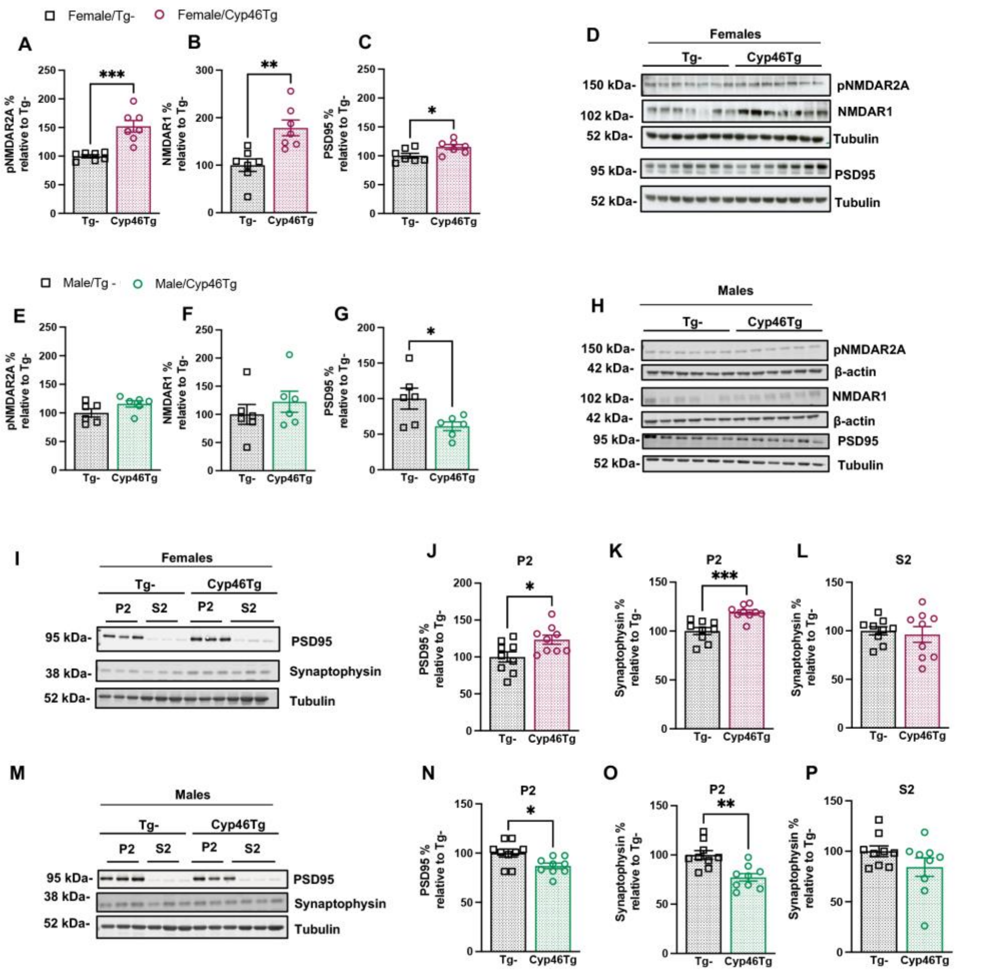

Fig. S2. **Synaptic protein levels increase in old Cyp46Tg female but decrease in Cyp46Tg males**. **A-C)** Immunoblotting analysis of pNMDAR2A, NMDAR1and PSD95 in hippocampi from Cyp46Tg and Tg- female mice (unpaired t test P=0.0003, P=0.0017, P=0.0270). **D)** Representative blots of synaptic proteins in female mice, Adapted data from (4) **E-G)** Western blot analysis of pNMDA2A, NMDAR1 and PSD95 protein levels from hippocampus homogenates from Cyp46Tg and Tg- male mice (unpaired t test P=0.0353)**. H)** Representative blots of synaptic proteins in male mice. **I)** Representative blots of PSD95 and Synaptophysin in synaptosome enriched fraction (P2) and cytosolic fraction (S2) from Cyp46Tg and Tg- female mice. **J-K)** Densitometric analysis of PSD95 and synaptophysin in Cyp46Tg females compared to Tg- mice (unpaired t test, P=0.0258 and P=0.0004) **L)** Synaptophysin in the S2 fraction of female mice. **M)** Representative blots from P2 and S2 fractions in Cyp46Tg males. **N-O)** PSD95 and Synaptophysin immunoblotting analysis of P2 fraction in Cyp46Tg male mice compared to Tg- (unpaired t test, P=0.0179 and P=0.0014) **P)** Synaptophysin in the S2 fraction of male mice. Data are shown as mean ± SEM of immunoreactivity (OD × area of the band) and normalized by housekeeping protein β-actin and tubulin. N=6-10 mice group/sex * P< 0.01 **P<0.001

Fig. S3

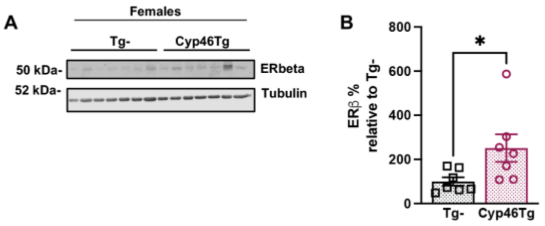

Fig S3. Levels of ERβ are elevated in hippocampus of old Cyp46Tg females. A) Representative blots of ERβ and tubulin in 18 months old Cyp46Tg and Tg- female mice. **B)** Densitometric analyses of ERβ normalized by tubulin in Cyp46Tg compared to Tg- female mice (unpaired t test, P=0.0386). Data are shown as mean ± SEM. N=7 mice/group. * P< 0.01

Fig. S4

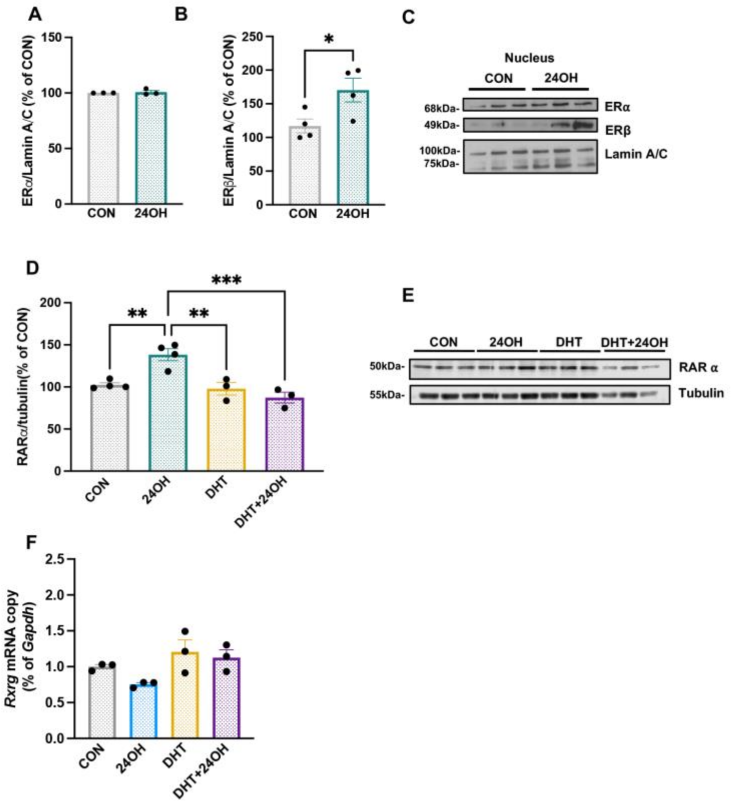

Fig S4. ***In vitro*** **treatments with 24OH and DHT+24OH. A-C)** Western blot analysis and representative blots of nuclear isolated ERα and ERβ protein levels normalized by lamin A/C levels in primary cultured neurons treated with 24OH (unpaired t test P=0.0349 for ERβ). **D, E)** Immunoblotting analysis and representative blot of RARα levels normalized by tubulin in hippocampal neurons treated with 24OH, DHT and 24OH+DHT (One-way ANOVA P=0,0006 followed by Tukey's multiple comparisons test: con vs 24OH P=0.005, 24OH vs DHT P=0.0038, 24OH vs 24OH+DHT P=0.0007) **F)** mRNA levels of *Rxrg* after 24OH treatment. Data are shown as mean ± SEM. N=3-4

independent experiments, performed in duplicates or triplicates. * P< 0.01 **P<0.001 ***P<0.0001.

Fig. S5

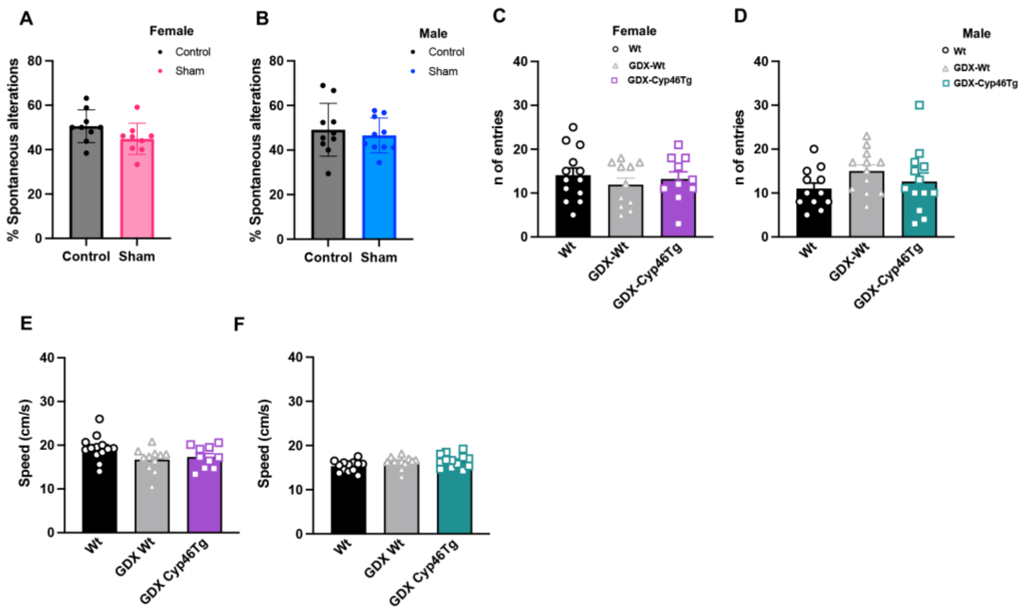

Fig S5. Cognitive and functional outcomes in gonadectomized mice. A-B) % of spontaneous alternations in the Y maze in non-operated (control) and sham operated female and male mice respectively **C-D)** number of entries in the Y maze test in Wt, GDX-Wt and GDX-Cyp46Tg female and male mice respectively. **E-F)** velocity during the learning phase of MWM test in Wt, GDX-Wt and GDX-Cyp46Tg female and male mice respectively. Data are shown as mean ± SEM.

Fig. S6

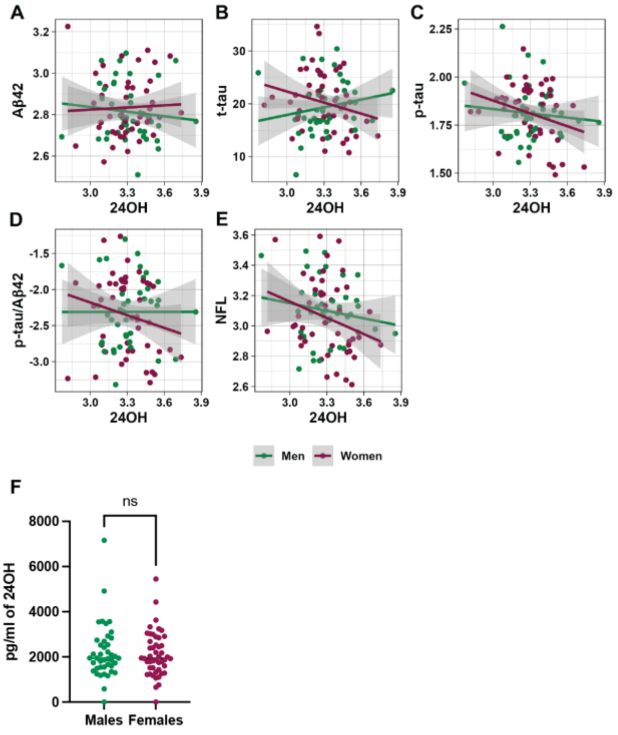

Fig S6. 24OH associations with AD biomarkers. A-E) Scatterplots depicting linear regression models with 24OH and Aβ42, t-tau, p-tau, p-tau/Aβ42 and NFL in men and

women separately **F)** CSF 24OH levels in in women and men from the memory clinic cohort**.**

Table S1

**Demographics**	**SCI**	**MCI**	**AD**	***P* value**
Age, years	62.4 (4.38)	65.6 (7.48)	68.2 (7.86)	0.005**
Female	60%	50%	50%	0.668
APOE e4 carrier[a]	31%	71%	76%	0.077
**Cognition**				
MMSE score[b]	28.1 (2.0)	27.7 (2.1)	23.2 (5.6)	<0.0001***
**Core AD CSF biomarkers**				
Aβ42, pg/ml	988.5(217.6)	569.2 (60.7)	555.3 (128.7)	<0.0001***
t-tau, pg/ml	251.1 (95.1)	464.3 (219.8)	551.3 (215.1)	<0.0001***
p-tau, pg/ml	61.4 (27.9)	68.4 (23.3)	74.0 (21.4)	0.108
p-tau/Aß42	0.06 (0.03)	0.12 (0.05)	0.14 (0.05)	<0.0001***
**Chronic conditions**				
Cardiovascular[c]	3.3%	6.7%	0%	0.355
Autoimmune[d]	3.3%	6.7%	6.7%	0.809
Osteoarthritis	13.3%	13.3%	6.7%	0.638

Table S1. Summary of the CSF study population characteristics. Data are shown as unadjusted mean (SD), unless otherwise stated. *P* values were calculated by analysis of covariance (ANCOVA), adjusting for age for continuous variables or by chi square for categorical data. $^{*}P < 0.05$, $^{**}P<0.01$, $^{***}P<0.001$

[a]*APOE* e4 carrier data was available for 13 SCD, 17 MCI and 17 AD participants
[b]MMSE score was available for 29 SCD, 29 MCI and 26 AD participants
[c]Coronary heart disease in one SCD and 2 MCI participants
[d]Autoimmune conditions found in the cohort included: psoriasis in one SCD and two MCI participants, Crohn's disease and ulcerative colitis in two AD participants respectively.

Table S2.

	**Aβ42** β (p-value)	**t-tau** β (p-value)	**p-tau** β (p-value)	**p-tau/Aβ42** β (p-value)	**NFL** β (p-value)
All participants (N=53)					
24OH	-.028 (.732)	-.111 (.347)	-.212 (**.046**)	-.188 (.095**)**	-.352 (**.001**)

Table S2. Linear regression model adjusting for age, diagnosis and APOE genotype in 53 participants.
